# Editorial: New Insights in Diagnosing and Treatment of Glucose Disorders and Obesity in Children and Adolescents

**DOI:** 10.3389/fped.2021.786055

**Published:** 2021-11-18

**Authors:** Enza Mozzillo, Giulio Maltoni, Valentina Chiavaroli, Klemen Dovc, Marco Marigliano

**Affiliations:** ^1^Section of Pediatrics, Department of Translational Medical Science, Regional Center of Pediatric Diabetes, University of Naples Federico II, Naples, Italy; ^2^Pediatric Unit, IRCCS Azienda Ospedaliero-Universitaria di Bologna, Bologna, Italy; ^3^Neonatal Intensive Care Unit, Pescara Public Hospital, Pescara, Italy; ^4^Liggins Institute, University of Auckland, Auckland, New Zealand; ^5^UMC-University Children's Hospital Ljubljana, Ljubljana, Slovenia; ^6^Pediatric Diabetes and Metabolic Disorders Unit, University of Verona, Verona, Italy

**Keywords:** obesity, diabetes, childhood, technology, adolescence

## Introduction

The diagnosis and treatment of disorders of glucose metabolism and obesity in childhood have significantly changed over the last decades. The impact of new technologies for the management of type 1 diabetes (T1D) (1), the progress in genetic testing for rare forms of diabetes, hyperinsulinism, and obesity (2), and the relevance of secondary diabetes (such as in Cystic Fibrosis) (3) have a growing importance in the diagnostic and therapeutic approach of pediatric endocrinologists and diabetologists.

This Research Topic includes a collection of papers on relevant topics in Pediatric Endocrinology that could help clinicians in better understanding new findings in the endocrine and diabetes fields.

Disorders in glucose metabolism range from hypoglycemia to diabetes mellitus. In fact, in some rare diseases, a mutation in the same gene can cause both hypoglycemia and hyperglycemia, as highlighted in the systematic review by Casertano et al., who propose a novel comprehensive diagnostic flow chart. Diabetes mellitus ranges from T1D, an autoimmune disease requiring insulin, to monogenic diabetes, rare and not necessarily needing insulin, and to secondary diabetes, usually treated with insulin at the onset.

A proper and early diagnosis of diabetes in the childhood and an appropriate therapeutic approach can have a relevant impact on the clinical and psychological outcomes. Peng et al. show that diabetic ketoacidosis (DKA) prevalence was unchanged in a large regional center of China. The authors underline that the increasing awareness of this condition in the community and among primary care physicians could lead to earlier diagnosis, and therefore reduce the rates of DKA at presentation.

Chiesa and Marcovecchio report that vascular complications and the associated mortality remain a major issue for youth with T1D. Thus, it is essential the awareness about their prevalence and the importance of their early prediction and prevention in order to improve the long-term prognosis of youth with T1D. However, cardiometabolic diseases and excess adiposity are strong predictors of morbidity and mortality in children across the spectrum of glucose disorders. Chung et al. show that certain Nuclear Magnetic Resonance derived biomarkers are useful to predict the cardiometabolic risk in youth with dysglycemia.

In order to prevent the cardiovascular complications of T1D, literature has given growing attention to the efficacy of technologies that allow to reach treatment targets for glucose control. Fuchs and Hovorka underline that new technologies, such as *closed-loop* insulin delivery systems, are transforming diabetes management by improving glycaemic control and quality of life in children and young people. Franceschi et al. show in a systematic review of literature that, even though the new technologies have demonstrated to improve metabolic control, it is fundamental to point out to the families the peculiarities of the different devices. On one side Continuous Glucose Monitoring (CGM) allows a tighter metabolic control with less hypoglycemic events compared to intermittent scanning (isCGM); on the other side, isCGM seems to have greater benefits on psychological outcomes. Troncone et al. contribute to the scientific debate on the psychological benefits for patients and their caregivers after participating in a diabetes camp, by examining potential changes in psychological measures of youths' psychosocial adjustment and perception of diabetes, and self-efficacy in disease management.

Over the last decades the diagnosis and management of pediatric monogenic diabetes (4, 5) has substantially improved, thanks to the advanced molecular biology techniques. In their papers Ngoc et al. and Di Iorio et al. underline that the correct genetic diagnosis improves treatment outcomes and prognosis of neonatal diabetes and Wolfram syndrome, respectively. In the latter it is shown that the clinical phenotype of Wolfram syndrome may include optic neuropathy and not only optic atrophy. Often, there is no specific relationship between the mutation and the clinical symptoms in some forms of monogenic diabetes, as stressed by Zhao et al. The authors firstly report EIF2AK3 mutations causing Wolcott-Rallison syndrome, in which diabetes is present.

Whether early detection and treatment of pre-diabetes may contribute to improve the clinical course of secondary diabetes, such as Cystic Fibrosis Related Diabetes, is debated in the systematic review of Mozzillo et al.. The authors underline that early diagnosis and prompt initiation of insulin therapy could have beneficial effects on clinical outcomes of patients with Cystic Fibrosis and pre-diabetes.

Obesity is a major concern worldwide as it can dramatically affect patient's quality of life, particularly among children and adolescents. Its etiology varies from idiopathic to syndromic, up to the rarest monogenic form. Alterations in the mechanism of polyphagia prevail in the forms of obesity secondary to gene mutations, as evidenced by Gregoric et al., who describe two cases of pro-opiomelanocortin (POMC) deficiency. Focusing on idiopathic obesity, the most common type in the pediatric population, special attention deserves the association with metabolic syndrome (MS). Individuals living with MS may show hypertension, diabetes, dyslipidemia, and are at risk of cardiovascular complications. Thus, it is very important to identify those at higher risk already in the pediatric age group. Zhang et al. demonstrate that relative children's lipid accumulation product is an effective indicator for predicting MS, while Yang et al. highlight that C1q is positively associated with obesity and components of MS. In addition, Rahman et al. show an association between insulin-like growth factor binding proteins (IGFBPs) with metabolic homeostasis in adolescents, and suggest its potential use as a biomarker for obesity.

Treating pediatric obesity is not always successful, and which is the most efficient approach is still discussed. Of note, severe obesity is associated not only with physical but also psychological complications, particularly in adolescence. Klemenčič et al. suggest that a reversible bariatric surgery approach led to improvements of psychological factors in adolescents with severe obesity.

We hope that this Research Topic will provide a valuable resource for the diagnosis and therapy of glucose metabolism disorders and the different etiologic types of obesity in pediatric age.

## Author Contributions

EM, GM, VC, KD, and MM wrote manuscript and made a substantial, direct and intellectual contribution to the work. All authors have approved the submission of the final version of this manuscript for publication, and have agreed to be accountable for all aspects for the work.

## Conflict of Interest

The authors declare that the research was conducted in the absence of any commercial or financial relationships that could be construed as a potential conflict of interest.

## Publisher's Note

All claims expressed in this article are solely those of the authors and do not necessarily represent those of their affiliated organizations, or those of the publisher, the editors and the reviewers. Any product that may be evaluated in this article, or claim that may be made by its manufacturer, is not guaranteed or endorsed by the publisher.
